# The positive impact of rituximab on the quality of life and mental health in patients with pemphigus

**DOI:** 10.1016/j.jdin.2022.01.004

**Published:** 2022-03-01

**Authors:** Hanan Rashid, Mila Poelhekken, Joost M. Meijer, Maria C. Bolling, Barbara Horváth

**Affiliations:** University of Groningen, University Medical Center Groningen, Department of Dermatology, Center of Blistering Diseases, European Reference Network-Skin Member, Groningen, Netherlands

**Keywords:** DLQI, mental health, pemphigus, quality of life, rituximab, TABQOL

*To the Editor:* Pemphigus is a group of chronic autoimmune blistering diseases affecting skin and mucosa with a huge impact on the quality of life (QOL).^1^, ^2^, ^3^ Although the efficacy and safety of rituximab in pemphigus are repeatedly shown, only few studies have investigated QOL and treatment burden. The objective of this study was to investigate the impact of rituximab on the general and treatment-specific QOL in pemphigus. This single-center observational study included patients with pemphigus treated with rituximab. Two infusions of 1000 mg of rituximab were administered within an interval of 2 weeks (M0 and M0.5), followed by 500 mg at months 6 (M6) and 12 (M12). Patient-reported outcome measurements were collected, including the following: Dermatology Life Quality Index (DLQI), visual analog scale in pain, Hospital Anxiety and Depression Scale, and Treatment of Autoimmune Bullous Disease Quality of Life (TABQOL). Repeated measures were performed using linear mixed models, with time as fixed effect. Overall, 47 patients with pemphigus were included, 29 with pemphigus vulgaris and 18 with pemphigus foliaceus (Table I). Table II depicts the decrease in patient-reported outcome measurements scores. Both DLQI and TABQOL showed a decrease among M0, M0.5, M6, and M12 (n = 47, *P <* .001 and *P =* .001, respectively). For both, the decrease was most pronounced between M0 and M6 (DLQI, −100%, *P <* .001; TABQOL, −36.4%, *P <* .001) and between M0 and M12 (DLQI, −100%, *P <* .001; TABQOL, −45.4%, *P =* .002). The number of patients in the DLQI category “no/minimal impairment” increased from 13 to 24 (+84.6%) between M0 and M6 and from 11 to 24 (+118.2%) between M0 and M12. The number of patients in the category “severe impairment” decreased from 10 to 0 (−100%) between M0 and M6 and 11 to 1 (−90.9%) between M0 and M12. The number of patients in the TABQOL category “low impact” increased from 10 to 16 (+60.0%) between M0 and M6 and from 10 to 17 (+70.0%) between M0 and M12. The number of patients in the category “high impact” decreased from 8 to 4 (+50.0%) between M0 and M6 and 8 to 2 (−75.0%) between M0 and M12. Hospital Anxiety and Depression Scale anxiety decreased among M0, M0.5, M6, and M12 (n = 47, *P =* .035), specifically between M0 and M12 (−50.0%, *P =* .002). Hospital Anxiety and Depression Scale depression showed no significant decrease during the rituximab treatment (n = 47, *P =* .378). Visual analog scale pain decreased among M0, M0.5, M6, and M12 (n = 47, *P =* .018), in particular between M6 and M12 (−58.1%, n = 34, *P =* .028) and between M0 and M12 (−60.6%, n = 31, *P =* .043). In this study, we showed the positive effect of rituximab on patients’ well-being, QOL, and treatment-specific QOL during rituximab treatment. The improvement in DLQI and TABQOL was observed after 6 and 12 months, proving the short-term and long-term effects of the first 2 infusions. The observed decline in the anxiety scores during treatment may contribute to the positive effect of rituximab. Contrarily, no improvement in depression was observed, possibly because it is a result of a combination of long-term non–disease-related factors and disease burden. A limitation of this study is the absence of the assessment of patient-reported outcome measurements in patients with pemphigus treated with other systemic therapies.^4^^,^^5^

**Table I:** Demographic and clinical characteristics of the patients with pemphigus treated with rituximab

Characteristics	Median (IQR) or mean (SD) or n (%)
Total number of patients with pemphigus	47 (100)
Age at M0, years	62.0 (IQR, 21.0)
Sex	
Female	28 (59.6)
Male	19 (40.4)
Pemphigus subtype	
PV	29 (61.7)
Mucosal involvement	9 (31.0)
Mucocutaneous involvement	20 (69.0)
PF	18 (38.3)
DSG1 titer at M0	245.9 (SD, 501.5)
DSG3 titer at M0	200.6 (SD, 624.5)
Time between diagnosis and M0, months	7.0 (IQR, 52.0)
Rituximab naive∗	35 (74.5)
PDAI activity score at M0, /250	10.0 (IQR, 11.5)
Concomitant use of systemic oral prednisone at M0	27 (57.4)

*DSG*, Desmoglein; *IQR*, interquartile range; *M0*, first infusion of 1000 mg of rituximab at month 0; *PDAI*, pemphigus disease area index; *PF*, pemphigus foliaceus; *PV*, pemphigus vulgaris; *SD*, standard deviation.

∗ Patients who have not received prior rituximab infusions.

**Table II:** Change in the median scores of patient-reported outcome measures during treatment with rituximab in patients with pemphigus

PROMs	M0 (median; IQR)	Estimate of fixed effects∗ (95% CI)	M0.5 (median; IQR)	Estimate of fixed effects∗ (95% CI)	M6 (median; IQR)	Estimate of fixed effects∗ (95% CI)	M12 (median; IQR)	% decrease M0-M6	% decrease M0-M12
DLQI (n = 47)	4.0; 10.0	4.9 (2.9-6.9)	3.0; 8.0	4.1 (2.3-5.8)	0.0; 3.0	0.4 (−0.7 to 1.6)	0.0; 2.0	100†	100†
TABQOL (n = 47)	11.0; 13.3	4.9 (2.3-7.5)	8.0; 11.3	3.9 (1.2-6.7)	7.0; 11.0	0.7 (−1.4 to 2.8)	6.0; 8.8	36.4†	45.4†
VAS pain (n = 47)	3.3; 16.5	7.0 (−0.5 to 14.4)	5.4; 13.4	7.4 (2.8-11.9)	3.1; 12.8	2.7 (−1.7 to 7.2)	1.3; 4.7	6.1	60.6†
HADS anxiety (n = 47)	4.0; 7.0	2.1 (0.7-3.5)	4.0; 7.0	1.9 (0.5-3.3)	4.0; 5.5	0.8 (−0.3 to 1.8)	2.0; 5.0	25.0	50.0†
HADS depression (n = 47)	3.0; 6.0	0.5 (−0.7 to 1.8)	3.0; 6.0	0.2 (−1.1 to 1.5)	3.0; 3.5	−0.2 (−1.1 to 0.7)	2.0; 5.5	0	17.4

*DLQI*, Dermatology Life Quality Index; *HADS*, Hospital Anxiety and Depression Scale; *IQR*, interquartile range; *M0*, first infusion of 1000 mg of rituximab at month 0; *M6*, third infusion of 500 mg of rituximab at month 6; *M12*, fourth infusion of 500 mg of rituximab at month 12; *PROMs*, Patient-reported Outcome Measurements; *TABQOL*, Treatment of Autoimmune Bullous Disease Quality of Life; *VAS*, visual analog scale.

∗ The reported values of the estimates of fixed effects represent the change of scores at M0, M0.5, and M6 in comparison with M12.

† *P* ≤ .05.

## Conflicts of interest

Dr Horváth reports fees from Janssen-Cilag (Advisory Boards, Educational Grants, Consultations, and Investigator Initiative Studies), AbbVie (Advisory Boards, Educational Grants, Consultations, and Investigator Initiative Studies), Novartis Pharma (Advisory Boards, Consultations, and Investigator Initiative Studies), UCB Pharma (Advisory Boards and Consultations), Leo Pharma (Consultations), Solenne B.V. (Investigator Initiative Studies), Celgene (Consultations and Investigator Initiative Studies), Akari therapeutics (Consultations and Investigator Initiative Studies), Philips (Consultation), Roche (Consultation), Regeneron (Consultation), and Sanofi (Consultation). Drs Rashid, Poelhekken, Meijer, and Bolling have no conflicts of interest to declare.
